# Neutrophils in Tuberculosis: Cell Biology, Cellular Networking and Multitasking in Host Defense

**DOI:** 10.3390/ijms22094801

**Published:** 2021-04-30

**Authors:** Rachana R. Borkute, Sören Woelke, Gang Pei, Anca Dorhoi

**Affiliations:** 1 Institute of Immunology, Friedrich-Loeffler-Institut, 17493 Greifswald-Insel Riems, Germany; rachanaramesh.borkute@fli.de (R.R.B.); soeren.woelke@fli.de (S.W.); gang.pei@fli.de (G.P.); 2 Faculty of Mathematics and Natural Sciences, University of Greifswald, 17489 Greifswald, Germany

**Keywords:** neutrophils, *Mycobacterium tuberculosis*, tuberculosis, cell biology, pathology

## Abstract

Neutrophils readily infiltrate infection foci, phagocytose and usually destroy microbes. In tuberculosis (TB), a chronic pulmonary infection caused by *Mycobacterium tuberculosis* (*Mtb*), neutrophils harbor bacilli, are abundant in tissue lesions, and their abundances in blood correlate with poor disease outcomes in patients. The biology of these innate immune cells in TB is complex. Neutrophils have been assigned host-beneficial as well as deleterious roles. The short lifespan of neutrophils purified from blood poses challenges to cell biology studies, leaving intracellular biological processes and the precise consequences of *Mtb*–neutrophil interactions ill-defined. The phenotypic heterogeneity of neutrophils, and their propensity to engage in cellular cross-talk and to exert various functions during homeostasis and disease, have recently been reported, and such observations are newly emerging in TB. Here, we review the interactions of neutrophils with *Mtb*, including subcellular events and cell fate upon infection, and summarize the cross-talks between neutrophils and lung-residing and -recruited cells. We highlight the roles of neutrophils in TB pathophysiology, discussing recent findings from distinct models of pulmonary TB, and emphasize technical advances that could facilitate the discovery of novel neutrophil-related disease mechanisms and enrich our knowledge of TB pathogenesis.

## 1. Introduction

Tuberculosis (TB) is a disease caused by bacteria of the *Mycobacterium tuberculosis* complex (MTC) and affects multiple species. *Mycobacterium tuberculosis* (*Mtb*) is adapted to humans and the main etiologic agent of human TB. However, it is as yet inaccurately known and likely underestimated how strong zoonotic infections, e.g., caused by *M. bovis*, contribute to human TB [1]. TB still is among those infectious diseases causing most fatalities worldwide, reaching around 1.4 million deaths in 2019 [2], despite being preventable and curable. The ever-growing incidence of TB caused by drug-resistant bacteria increases this threat to global health. TB primarily affects the lung, albeit zoonotic infections often result in extra-pulmonary manifestations. The disease pathophysiology is complex: dynamic immune responses contribute to *Mtb* persistence in infected hosts in the absence of clinical disease (latent TB infection (LTBI)) and govern clinical manifestations as well as heterogeneous tissue pathology in active disease. There are hints that early immune events control the clearance of infection in TB resisters [3]. Mycobacteria encounter various cell types within the respiratory parenchyma, yet *Mtb* is primarily contained in phagocytes, with macrophages being extensively studied as *bona fide* host cells harboring bacilli [4].

Towards the infection site, neutrophils are promptly recruited. This has been shown in murine [5,6,7,8], non-human primate [9] and cattle [10,11] models of TB, indicating that neutrophils have a prominent role in TB pathogenesis. They are abundant, highly motile innate immune cells that readily phagocytose microbes and usually destroy them via reactive oxygen species (ROS), proteases and antimicrobial peptides (AMPs). These antimicrobial molecules, however, can also indiscriminately damage host tissue. Neutrophils are abundant in TB lesions [12,13,14] and represent the most abundant cell type containing bacilli in the sputum of TB patients [15]. Thus, neutrophils likely mediate *Mtb* transmissibility and access to new hosts [16,17,18] and in addition alter the fate of the bacteria in permissive macrophages [19]. The deleterious roles of neutrophils for TB outcome have been well documented in experimental models [20,21,22,23,24] and neutrophil abundances in blood and bronchoalveolar fluid correlate with the severity of TB in humans [25,26,27,28,29]. Neutrophils could supply *Mtb* with nutrients and cause tissue damage by means of their cellular storage of antimicrobials. They have, however, been assigned protective roles in *Mtb*-infected rhesus macaques [30], but an inverse correlation between neutrophil numbers and the risk of TB has been established in humans [31]. Undoubtedly, such contradictory findings suggest the versatile roles of neutrophils and indicate that many aspects of their biology in TB remain scantly addressed.

This review discusses the interaction of neutrophils with mycobacteria, i.e., host receptors and intracellular pathways that control host cell activation and cell fate. In the context of pulmonary TB, the interaction of neutrophils with myeloid and parenchyma cells and the consequences for TB progression, bacterial dissemination and resolution of inflammation are reviewed. Advances in neutrophil biology provided by distinct experimental animal models for pulmonary TB are critically discussed and the pros and cons of the models are described. Finally, we formulate open questions regarding the cellular microbiology of *Mtb*-infected neutrophils and emphasize how novel technologies could help fill the gaps in the current understanding of neutrophil roles in TB pathophysiology. Neutrophil-targeted therapies for TB have been covered by recent reviews [32,33] and will not be the focus of this work.

## 2. The Infected Neutrophil: Events within and its Fate

**Figure 1:**
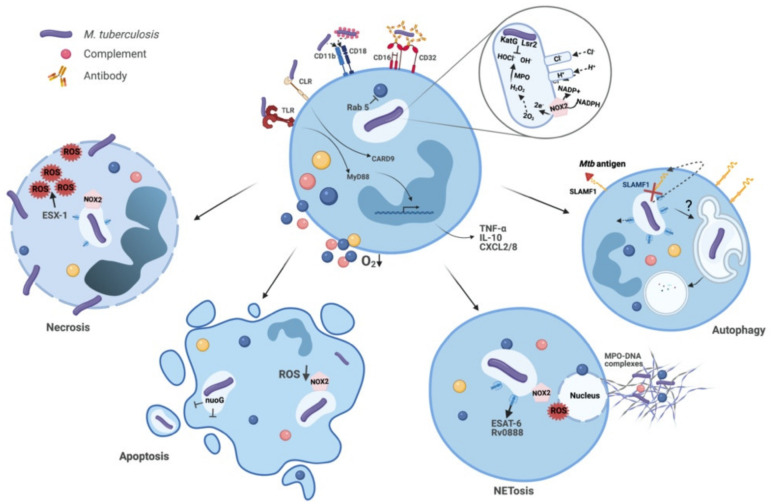
Phagocytosis of mycobacteria and neutrophil fate post-infection. Opsonic, i.e., Fc receptors (FcγRIII/FcγRII, CD16/CD32) and complement receptors (CR3, CD11b/CD18), and non-opsonic, i.e., C-type lectin receptors (CLR), receptors control the internalization of mycobacteria. Neutrophil activation, including the release of cytokines and chemokines as well as degranulation, is in addition triggered by Toll-like receptors (TLR) and modulated by hypoxia. Phagosomal recruitment of the nicotinamide adenine dinucleotide phosphate oxidase (NADPH oxidase, NOX2) and the production of reactive oxygen species (ROS) do not eliminate *Mycobacterium tuberculosis* (*Mtb*). Abundant ROS rather cause neutrophil necrosis and the formation of neutrophil extracellular traps (NETs), whereas mycobacterial factors restrict apoptosis and autophagy. Abbreviations: CARD9, caspase recruitment domain-containing protein 9; CD, cluster of differentiation; CXCL, (C-X-C motif) ligand; ESAT-6, 6 kDa early secretory antigenic target; ESX-1, 6 kDa early secretory antigenic target secretion system 1; IL, interleukin; KatG, catalase-peroxidase; Lsr2, nucleoid-associated protein of *Mtb*; MPO, myeloperoxidase; MyD88, myeloid differentiation primary response 88; nuoG, operon in *Mtb*; O_2_, oxygen; SLAMF1, signaling lymphocytic activation molecule family member 1; TNF, tumor necrosis factor. *Image created with BioRender.com*.

As classical phagocytes, neutrophils efficiently phagocytose mycobacteria and a series of cellular events are triggered upon, or following, the internalization of TB bacilli. We review the molecular mechanisms of phagocytosis, phagosomal maturation, degranulation, autophagy, the generation of neutrophil extracellular traps (NETs) and the cell death patterns detected in neutrophils during mycobacterial infection (Figure 1).

### 2.1. Mtb Recognition and Phagocytosis

Neutrophils express a variety of receptors on their surface for pathogen recognition and further downstream responses, such as chemotaxis, immune signaling and phagocytosis. They express G-protein-coupled receptors such as formylpeptide receptors (FPRs), chemoattractant (e.g., leukotriene B4 receptor 1 (BLT1), platelet-activating factor (PAFR), complement component 5a receptor (C5aR)) and chemokine receptors (e.g., CXCR1, CXCR2) that regulate cell migration [34]. Neutrophils also express several Fc-receptors (e.g., FcγRI, FcγRIIA and FcγRIII) and complement receptors (e.g., CR3) that initiate the phagocytosis of opsonized microbes [34,35], as well as pattern-recognition receptors (PRRs), including Toll-like receptors (TLRs), C-type lectin receptors (CLRs), scavenger receptors (SRs), and NOD-like receptors (NLRs), which trigger signaling cascades and regulate immune and inflammatory responses. PRRs sense structurally conserved microbial moieties. The *Mtb* cell wall contains several glyco- and sulpho-lipids, i.e., lipomannan (LM), lipoarabinomannan (LAM), mannosylated lipoarabinomannan (ManLAM), phthiocerol dimycocerosate (PDIM), mycolic acids, specifically trehalose-6,6-dimycolate (TDM) and sulpholipids (SL), which are selectively detected by neutrophils. The acidic lipid SL-1 activates the guanine nucleotide-binding protein Rac2 and causes diacylglycerol generation and calcium mobilization in human neutrophils [36]. LAM sensing by TLR2 results in the activation of the p38 mitogen-activated protein kinase (MAPK) for pro- and anti-inflammatory cytokine release. However, unlike the synthetic TLR2 agonist Pam_3_CSK_4_, *Mtb* LAM recognition does not mobilize granule exocytosis in human neutrophils [37]. TLR2 also recognizes mycobacterial lipopeptides, i.e., the 19 kDa lipoprotein [38], as well as LM [39] and phosphoinositol mannoside (PIM) [40]. The sensing of these *Mtb* moieties by neutrophils triggers cell activation. Mycobacterial glycolipids are also sensed by several CLRs. Mincle expressed by neutrophils recognizes TDM and regulates cytokine (TNF-α, IL-6, CCL2) production [41]. The C-type lectin receptor CLECSF8/CLEC4D/MCL, which is highly expressed on human neutrophils [42], senses TDM in association with the FcRγ [43]. Dectin-1 is also involved in cytokine release by neutrophils; however, the exact mycobacterial ligand binding to this CLR remains undefined [44]. Mycobacteria co-trigger the CLR/Dap-12/Syk/Card9 and the TLR/MyD88 pathways for the production of inflammatory as well as regulatory cytokines, i.e., IL-10, in murine neutrophils [44,45]. The outcome of these innate signaling pathways in human cells is likely restricted to inflammatory mediators, and likely also priming and degranulation, as noted above for TLR2 mycobacterial agonists. Propensity to release IL-10 is debatable for human neutrophils [46]; however, it has been demonstrated for cells from *Mtb*-infected macaques, both intralesional and ex vivo [47].

The phagocytosis of mycobacteria by neutrophils is mediated by opsonic receptors, i.e., CRs and FcRs, and non-opsonic receptors, notably CLRs. Several lectins relevant to *Mtb* uptake in macrophages, i.e., the mannose receptors, are not present in human neutrophils [48]; however, the involvement of few CLRs in mycobacterial phagocytosis by neutrophils has been established. CLECSF8 controls *Mtb* binding to neutrophils [49] and Dectin-1 may affect the internalization of *M. bovis* BCG aggregates [50]. CR3 controls the phagocytosis of mycobacteria in different ways. CR3, also known as CD11b/CD18, Mac-1 or αMβ2 integrin, has two domains that recognize different epitopes of mycobacteria. The I-domain binds to complement the fragment iC3b [51] and the mycobacterial antigen 85C, whereas the lectin-like C-domain binds to mycobacterial LAM [52]. Phagocytosis of *M. kansasii* and *M. smegmatis* occurs through the binding of the bacilli to CR3 via glycosylphosphatidylinositol-anchored proteins [53] and implies relocation of the receptor in cholesterol-rich microdomains [54]. These events facilitate the engulfment of the bacilli by macrophages and neutrophils under non-opsonic conditions [53,55]. The phenolic glycolipid 1 (PGL-1) from *M. leprae* also binds to CR3 for non-opsonic entry into neutrophils [56]. The precise events at the human neutrophil membrane essential for mycobacterial uptake, linking CR3 and cell membrane cholesterol, have recently been unveiled. The glycosphingolipid lactosyl ceramide, which is enriched in the lipid rafts, binds to LAM and thereby mediates the phagocytosis of mycobacteria regardless of their pathogenicity [57]. In the case of virulent species, which contain mannose-capped LAM, this moiety binds to lactosyl ceramide through its α1,2-mannose structures within the LAM mannan core. This interaction modulates signaling rather than bacilli engulfment through reorganization of the glycosphingolipid in the phagosome membrane [57]. Thus, cholesterol and lactosyl ceramide partitioning in membrane microdomains, their organization and the localization of receptors, notably CRs, enable the uptake of mycobacteria by neutrophils.

### 2.2. Phagosome Maturation and Evasion by Mtb

Nascent phagosomes containing microbial cargo undergo a maturation process that differs in neutrophils compared to macrophages. Phagosome maturation is best characterized in macrophages, in which nascent phagosomes sequentially fuse with early and late endosomes and finally with lysosomes, resulting in phagolysosomes. During this process, the phagosomal pH decreases dramatically, which facilitates enzymatic degradation of the cargo. The NADPH oxidase NOX2, and in murine cells inducible nitric oxide synthase (NOS2), which produces reactive nitrogen species (RNS), is recruited to early phagosomes, leading to phagosomal ROS and RNS production and bactericidal activity [58,59,60]. Neutrophils lack the classical endocytic pathway and have few lysosomes. Nascent phagosomes fuse with granules during the maturation process. The contents from primary and secondary granules are delivered to phagosomes [61,62] and IgG-opsonization increases the fusion events, suggesting dependency on receptor-mediated signaling [62]. Fusion occurs shortly after the sealing of phagosomes [63]. The cytoskeleton components, e.g., microtubules [64], are likely involved in promoting the fusion of granules during phagosome maturation in neutrophils [65]. However, none of the Rab GTPases, the central regulators controlling membrane trafficking, have been identified in the phagosomes of neutrophils [65]. Whilst granule exocytosis necessitates cytosolic calcium [66] and this is required for the fusion of primary granules with phagosomes containing IgG-opsonized zymosan or nascent phagosomes [64,67], calcium appears obsolete for fusion events of sealed phagosomes. Thus, different mechanisms regulating phagosome-granule fusion exist [67] and precise dynamics and molecular regulators remain to be clarified.

Pathogenic mycobacteria have developed various strategies to manipulate phagosome maturation in macrophages. Similarly, the fusion of primary granules with phagosomes in human neutrophils is inhibited by pathogenic mycobacteria (*M. kansasii*, *M. avium* and *Mtb*) and left unchanged by non-pathogenic mycobacteria. The membrane association of the NADPH oxidase components p47phox and p67phox and the production of superoxide similarly occur irrespective of the pathogenicity of the mycobacteria [68,69,70]. Rab5, which controls phagosome fusion with early endosomes, is aberrantly retained in *Mtb*-containing phagosomes in macrophages, leading to the arrest of phagosome maturation [71]. In neutrophils, Rab5 and its effector Syntaxin-4 are also retained much longer in *Mtb*-containing phagosomes. Hence, the prolonged association of Rab5 with phagosomes in neutrophils could explain the inhibition of fusion between primary granules and *Mtb*-containing phagosomes [69], yet experimental proof is lacking.

### 2.3. Antimicrobial Mechanisms and the Fate of Intracellular Mtb

The microbial killing machinery of neutrophils encompasses ROS and AMPs, as well as various hydrolases and enzymes. In addition, neutrophils release extracellular traps (NETs) to entangle and destroy microbes within.

#### 2.3.1. Granular Enzymes and AMPs

Neutrophil antimicrobial enzymes are stored in granules [72]. Certain proteins from primary and secondary granules of human neutrophils show antimycobacterial activities against *M. smegmatis*, *M. bovis* BCG and *Mtb in vitro* [73]. Yet, all these molecules need to work in concert to restrict bacillary replication. The primary azurophilic granules contain serine proteases, including neutrophil elastase (NE), cathepsin G (catG) and proteinase 3, as well as α-defensins, and the oxidant-producing and membrane-permeabilizing enzymes myeloperoxidase (MPO) and lysozyme, respectively, which play a crucial role in microbial killing [72]. A study by Andersson et al. provides evidence as to how dying apoptotic neutrophils, containing active MPO, suppress *Mtb* growth in infected macrophages [74]. The antibacterial activity of MPO has been observed also for *M. leprae* [75]. CatG and NE are important for controlling mycobacterial replication during the early stages of infection in mice [76]. Cathelicidin LL-37, also known as human cationic antimicrobial peptide, is the only cathelicidin identified in humans. During *Mtb* infection, neutrophils produce LL-37 and restrict bacillary growth, which indicates that the peptide has antimicrobial activity [31,77]. α-Defensins are small (3–4 kDa) cysteine-rich cationic peptides with different antibacterial spectra, which are released into phagosomes at a very high concentration upon fusion with primary granules [78]. Within this class, human neutrophil peptide-1 to 3 (HNP-1 to 3) are the major antimicrobial members. HNP-1 exhibits anti-mycobacterial activity *in vivo* and in vitro [79]. It also shows a synergistic effect with anti-tuberculous chemotherapy in vitro [80]. In evolution, dramatic variations in these peptides in different mammals have taken place. In swine, cattle and mice neutrophils are deficient of α-defensins. However, in cattle an expansion of β-defensins (at least 58 members) has been identified, and many of them are produced by neutrophils [81,82]. Anti-mycobacterial activity has been reported for the mature bovine neutrophil β-defensins (mBNBD) 4 and 5 [83,84].

The secondary and tertiary granules contain lactoferrin and gelatinase, respectively [72], as well as lipocalins. Unlike the azurophilic granules, which are released into the phagosome, the secondary and tertiary granules are directly mobilized to the cell surface prior to the bacterial encounter and their release threshold is lower. Lactoferrin displays broad antibacterial activities by sequestering iron [85]. Lactoferrin treatment results in increased uptake of *M. bovis* BCG and elevated apoptosis in vitro [86]. The oral administration of lactoferrin to mice enhances IFN-γ-mediated *Mtb* killing by macrophages in a nitric oxide (NO)-dependent manner [87]. Lipocalin-2 is enriched in neutrophils [88] and by sequestering bacterial siderophores it limits bacterial acquisition of iron, consequently inhibiting bacterial growth [89]. Lipocalin-2 deficiency results in increased susceptibility to *M. bovis* BCG infection [90]. However, lipocalin-2 knockout mice have a lower *Mtb* load at early stages of infection only [91]. These discordant findings may be due to various kinetics of neutrophils in the two mycobacterial models, variable iron pools, or the distinct requirements for iron by the MTC members.

#### 2.3.2. ROS

Microbial internalization initiates a cascade of events, including the assembly of the NADPH complex on the phagosome membrane and the initiation of oxidative burst. Superoxide has been proposed as an antimicrobial fuel in neutrophils for a long time [92]. Indeed, ROS generation by NADPH oxidase is critical for host defense against distinct microbes [93,94]. The importance of the oxidative burst as an antimicrobial has been unambiguously proven by the discovery of chronic granulomatous disease (CGD). CGD patients exhibit mutations altering the NADPH oxidase and are susceptible to bacterial and fungal infections due to a low oxidative burst in neutrophils [94]. NADPH oxidase includes the catalytic subunit gp91phox, the transmembrane protein p22phox, the cytosolic subunits p40phox, p47phox and p67phox, and the nucleotide-binding proteins Rac2 [93]. The activated NADPH oxidase generates superoxide by transferring electrons to molecular oxygen. The superoxide dismutase catalyzes superoxide to hydrogen peroxide [92], which is converted into a toxic oxidant, hypochlorous acid (HOCl), and other reactive intermediates by MPO and thus the encountered pathogen is eliminated, as demonstrated by *Staphylococcus aureus* killing in vitro [72,95].

Pathogenic (*Mtb*) as well as non-pathogenic (*M. smegmatis*) mycobacteria trigger robust ROS release in human neutrophils [70]. However, various mycobacterial pathogen-associated molecular patterns (PAMPs) differ in their capacity to induce oxidative burst. Phenolic glycolipids (PGLs) from distinct mycobacteria differently induce the production of ROS. For instance, PGLs from *Mtb* and *M. kansasii*, which contain tri- and tetra-saccharides, respectively, trigger ROS in neutrophils, whereas PGLs from *M. bovis* BCG and *M. marinum*, which contain only a monosaccharide, do not induce ROS release [96]. The NADPH oxidase controls susceptibility to TB in humans; however, experiments in mice yield variable outcomes. Convincing evidence for the role of oxidative burst in the control of *Mtb* is provided by observations in CGD patients. These individuals have recurrent or disseminated TB. Macrophages from patients with mutations in the gp91phox subunit of the NADPH oxidase show compromised oxidative burst upon *M. bovis* BCG infection, suggesting that a functional NADPH oxidase is critical for proper protection against mycobacterial diseases [97,98,99]. Further, one of the NADPH subunits, gp91phox(Cybb), is associated with TB susceptibility in humans [98]. However, mice lacking gp91phox or gp47phox and thus resembling CGD do not phenocopy observations in humans [100]. Experimental infection of gp47phox knockout mice with *Mtb* led to susceptibility to TB; however, elevated bacterial replication occurred at initial disease stages, prior to the generation of acquired immunity [100]. X-linked CGD mice exhibit enhanced *Mtb* growth only in the lungs, but not in the spleen and liver [101]. The gp91phox-deficient mice display no defects in controlling *Mtb* burden early in infection [102]. At late stages of TB, gp91phox-deficient mice develop hyper-inflammation, with more neutrophils and higher IL-1β levels in the lungs and succumb more rapidly than wildtype mice. Recently, an elegant mouse study using gp91-deleted animals has unveiled unchanged *Mtb* burdens, and an increased accumulation of neutrophils in the lungs of the mutant mice due to inflammasome activation. These findings emphasize a role of ROS in disease tolerance [103]. Hence, gp91phox appears more prominent in promoting tolerance to TB and inhibiting inflammation rather than controlling *Mtb* replication by oxidative killing [103].

The unclear role of NADPH oxidase in limiting *Mtb* replication is possibly due to the ROS scavenging mechanisms employed by *Mtb* to escape from oxidative killing. KatG detoxifies ROS directly and is critical for the virulence of *Mtb* in mice [104]. Lsr2, a histone-like protein, binds to the DNA of *Mtb* and protects it against ROS damage [105]. Furthermore, the superoxide-detoxifying enzyme (SodA), an integral membrane protein (DoxX) and a predicted thiol-oxidoreductase (SseA), form a membrane-associated oxidoreductase complex (MRC) that alleviates oxidative damage by maintaining thiol homeostasis [106]. In line with these reports, *Mtb* escapes from killing by phagosomal ROS in neutrophils, and the activation of NADPH oxidase rather enforces necrotic cell death and the spread of infection to other cells [70].

#### 2.3.3. NETs

NETs are mesh-like structures consisting of decondensed chromatin, AMPs and granule proteins such as NE and MPO [107], and they are microbicidal, contributing to host defense against pathogens [108]. NETs can cause tissue damage if their formation is excessive or uncontrolled and hence this neutrophil defense strategy has been linked to autoimmune diseases [107]. MPO–DNA complexes, an indicator of NETs, are elevated in plasma from TB patients compared to LTBI and healthy individuals and correlate with *Mtb* burden [109]. Similarly, the plasma levels of nucleosomes and NE are elevated in TB patients [110]. NETs have also been reported in necrotic TB lesions [111], suggesting a role in TB pathogenesis. *Mtb* induces in vitro NETs in low-density granulocytes (LDGs), a specific subpopulation of neutrophils. Moreover, LDGs from TB patients spontaneously release abundant NETs in a process dependent on ROS generation [112]. *Mtb* triggers the formation of NETs in neutrophils from healthy donors and resists killing by NETs [113]. Intradermal [114] as well as aerosol infection [111,115] of rodents with *Mtb* induce the generation of NETs, which are, as demonstrated *in vitro*, unable to destroy bacilli within lesions [114]. Bacterial factors, including the ESAT-6 pore-forming secreted protein [116] and the Rv0888 secreted protein that have both nuclease and sphingomyelinase activities, induce NETs [117]. On the host side, type I interferon (IFN-I) has been involved in the generation of NETs and the exacerbation of TB pathology [111]. *Mtb*-induced NETs can be phagocytosed by macrophages, which subsequently release high amounts of IL-6, TNF-α, IL-1β and IL-10, possibly contributing to inflammation [118]. Thus, *Mtb* exploits NETs to cause immunopathology and eventually destroy tissue, thereby enabling its transmission. NETotic cells, just as necrotic neutrophils, could harbor *Mtb* with viability rates and particle sizes similar to those of free mycobacteria [17]. Moreover, by entangling but not killing the *Mtb*, neutrophils undergoing NETosis could generate mycobacterial aggregates that protect bacilli from environmental stress, increase their tenacity and facilitate successful transmission to close contacts.

The above-mentioned antimicrobial mechanisms, while effective against multiple microbes, have limited efficacy against *Mtb*. Concerning the intracellular fate of mycobacteria, evidence suggests that naïve human neutrophils fail to kill opsonized *Mtb* despite being activated. ROS rather causes necrotic cell death in neutrophils [70]. However, neutrophils are able to kill *Mtb* H37Ra, an attenuated strain of *Mtb*, in a calcium-dependent manner [119]. Other ex vivo studies show that neutrophils from CGD patients exhibit killing of *Mtb* comparable with neutrophils from healthy donors, suggesting a killing mechanism independent of the oxidative burst [120]. In line with these observations, TNF-α stimulates the killing of *Mtb* by human neutrophils partially dependent on α-defensins [121]. Upon *Mtb* exposure to human alveolar lining fluid, the intracellular killing by neutrophils is significantly increased. This could be due to alteration in the *Mtb* cell envelope by hydrolases contained in the alveolar fluid [122]. Conversely, another study has demonstrated that neutrophil depletion in whole blood results in 7.3-fold and 3.1-fold increases in *M. bovis* BCG or *Mtb*, respectively, supporting the anti-mycobacterial roles of neutrophils [31]. Additionally, antimicrobial peptides from neutrophils (lipocalin 2, LL-37, and HNP-1-3) at high concentrations display significant anti-tuberculous activities [31]. The discrepancies regarding the capacities of neutrophils to destroy *Mtb* may stem from the distinct antimicrobial features of the cells employed in the mentioned reports, different isolation and/or infection protocols, e.g., bacterial opsonization, the existence of variable populations of neutrophils and lastly cross-talks with other immune cells, such as macrophages, dendritic cells and T-cells, which contribute to the intracellular control of *Mtb*.

### 2.4. Degranulation and Tissue Pathology

Degranulation, a special form of exocytosis in neutrophils, consists of an active release of the granular content into the extracellular compartment. The degranulation kinetics of various granules differ substantially. Secretory vesicles are completely exocytosed, whereas only approx. 7% of primary granules are mobilized for exocytosis. Neutrophil degranulation helps to eliminate microbes, yet excessive degranulation may lead to tissue destruction and even disorders of the immune system. Signaling via integrins during neutrophil adhesion and/or the ligation of immune receptors, e.g., PRRs, FPRs and FcγRs, initiate the degranulation process.

Avirulent mycobacteria, such as *M. smegmatis*, do not trigger the degranulation of secretory, primary and secondary granules in human neutrophils, even upon microbial opsonization. However, opsonized *M. smegmatis* elicits a time- and presumably CR-dependent secretion of active matrix metalloproteinase 9 (MMP9), which is stored in tertiary granules [123]. Attenuated mycobacteria, such as *M. bovis* BCG, promote release of the TNF-related apoptosis-inducing ligand TRAIL/APO-2L, which is mainly present in the primary and secretory vesicles of human neutrophils [124]. *Mtb* induces MMP8 secretion in an AMPK-dependent manner. The MMP8 concentration in the sputum from TB patients correlates with the severity of the clinical disease, and it induces matrix destruction both in vitro and within lesions [16]. Interestingly, hypoxia increases the secretion of MMP8/9 and NE by *Mtb*-infected neutrophils, suggesting that particularly in oxygen-deprived environments, i.e., in granulomas, neutrophils could aggravate tissue pathology [125]. In human pulmonary TB, granulomas are hypoxic [126] and hence hypoxia modulates neutrophil biology and primes them for exacerbated tissue destruction. Strategies targeting neutrophils to restrict pulmonary damage in TB have already been envisaged [32].

### 2.5. Autophagy Regulation and Control of Inflammation

Autophagy facilitates the disposal of intracellular pathogens, and this process has been observed in infected neutrophils. Human neutrophils kill *Bacillus anthracis* and *Burkholderia pseudomallei* via autophagy [127,128] and the stimulation of this cell-autonomous process in neutrophil-like cell lines reduces the intracellular survival of adherent-invasive *E. coli* [129]. In macrophages, *Mtb* translocates from the phagosomes into the cytosol and subsequently the cytosolic bacteria are recognized by the autophagic machinery and targeted into autophagosomes for degradation [130]. Human neutrophils efficiently kill *M. abscessus*; however, the bactericidal activity is autophagy- and ROS-independent [131]. Recent data suggest that in human neutrophils the co-stimulatory molecule SLAMF1 increases autophagy during *Mtb* infection and bacilli subvert this pathway as TB patients show a lower abundance of SLAMF1 [132]. In mice, the deletion of the central autophagy regulator Atg5 in myeloid cells impairs survival and causes a high *Mtb* burden in tissue. Moreover, defective autophagy leads to excessive lung inflammation with increased neutrophil infiltration [133]. Intriguingly, mice deleted of other essential autophagy components, such as ULK1, ULK2, ATG4B, ATG3, ATG7, ATG14 and ATG16L, efficiently control *Mtb* replication suggesting an autophagy-independent role of Atg5 in murine TB. Neutrophil-specific *Atg5* deletion leads to increased TB susceptibility and exuberant neutrophil-associated inflammation. Thus, the hyper-susceptibility of *Atg5*-deficient mice is controlled by neutrophil-intrinsic mechanisms unrelated to autophagy defects [134]. Whether deletion of autophagy genes that impact granulopoiesis [135] alters the biochemical features or the phenotype of neutrophils and these changes subsequently affect inflammation remains to be elucidated.

### 2.6. Cell Death Patterns

The fate of infected neutrophils affects the survival of microbes within and also modulates local inflammation. The pathways governing myeloid cell death and its impact on TB outcome have been studied in great detail for macrophages [136]. Attenuated mycobacteria cause higher apoptosis rates, while virulent strains elicit negligible apoptosis but necrotic cell death [136,137]. In addition, the apoptotic vesicles from mycobacteria-infected macrophages stimulate cross-presentation and protect against TB in murine models [138,139]. Apoptosis has been observed in human neutrophils in vitro during infection with both attenuated and virulent mycobacteria [69,140,141,142]. Mycobacterial lipopeptides triggering p38 MAPK activation [141], or the induction of ROS by *Mtb* followed by the alteration of Bax/Bcl-x(L) expression and caspase-3 activation [69], regulate apoptotic cell death in human cells. More recently, evidence has been obtained that *Mtb* causes primarily necrotic cell death in neutrophils and that this process is controlled by the ESX-1 virulence factors, including ESAT-6 [116] on the bacterial side, and is conditioned by ROS production in host cells [19]. Necrotic death controlled by ESAT-6 and independent of caspase-1 and cathepsin B has also been reported in human macrophages [143]. In line with these observations, and supporting a host-protective role of apoptosis, TB susceptible mice show lower rates of neutrophil apoptosis and a positive correlation between neutrophil apoptosis and TB protection has been established [144]. Mechanistically, phagocytosis of *Mtb*-induced apoptotic neutrophils by macrophages dramatically increases the production of the proinflammatory cytokine TNF-α [145] and enhances its capacity to control intracellular *Mtb* growth possibly by boosting NLRP3 inflammasome activation [146]. Opposingly, necrotic neutrophils promote bacillary replication in macrophages [19] and in human whole blood, causing in addition immune suppression [147]. Besides the regulation of necrosis by ESX-1, *Mtb* employs virulence factors, notably gene products encoded in the nuoG operon, to restrict neutrophil apoptosis *in vivo*. *Mtb* ΔnuoG induces higher apoptosis in myeloid cells, including neutrophils, and more abundant Ag85B-specific CD4^+^ T-cells. This process is neutrophil-dependent and suggests that neutrophil apoptosis enhances adaptive immunity in TB [148]. Collectively, neutrophil apoptosis limits inflammation, enhances adaptive immunity and bystander neutrophil apoptosis may contribute to *Mtb* elimination. Virulent mycobacteria have developed several strategies to inhibit apoptosis and cause necrotic cell death and thereby facilitate their spread to new hosts.

## 3. The Networking Neutrophil: Dialogues with Impact on TB Outcome

**Figure 2:**
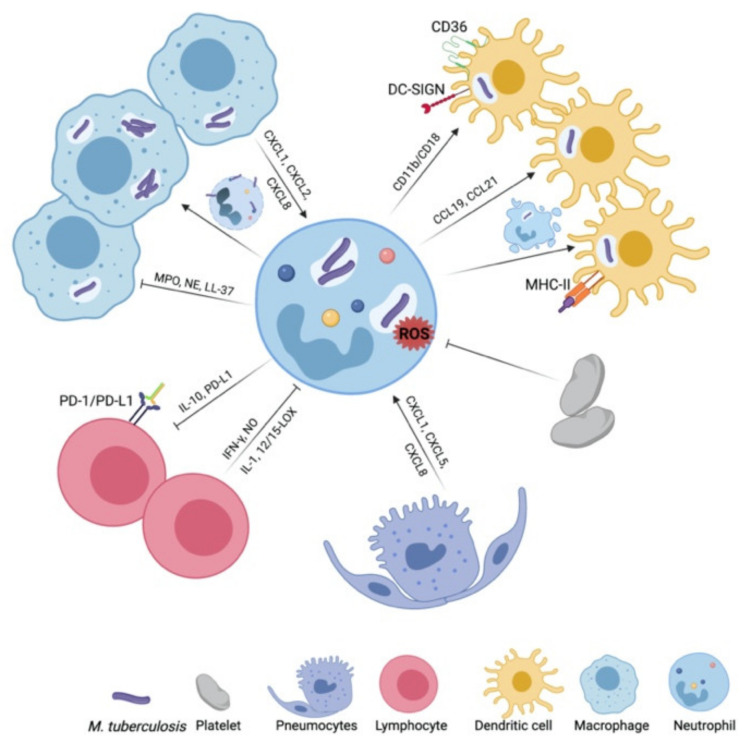
Neutrophil networking in tuberculosis (TB). Neutrophils and macrophages cross-talk in TB in multiple ways. Macrophages modulate neutrophil dynamics and neutrophils limit or facilitate the replication of *Mycobacterium tuberculosis* (*Mtb*) within macrophages. The networking of neutrophils with dendritic cells (DCs) promotes antigen presentation and cross-presentation. In a direct bidirectional cross-talk with T-cells, neutrophils restrict T-cell proliferation and cytokine production, whereas T-cells use IFN-γ to limit tissue accumulation and the *in situ* viability of neutrophils. In early TB, platelets alter ROS production in neutrophils and pneumocytes contribute to the recruitment of neutrophils to the *Mtb* infection site. Abbreviations: CD, cluster of differentiation; CCL, (C-C motif) ligand; CXCL, (C-X-C motif) ligand; DC-SIGN, dendritic cell-specific intracellular adhesion molecule-3-grabbing non-integrin; IL, interleukin; IFN, interferon; LL-37, cathelicidin antimicrobial peptide; LOX, lipoxygenase; MHC-II, major histocompatibility complex class II; MPO, myeloperoxidase; NE, neutrophil elastase; NO, nitric oxide; PD-1, programmed cell death protein 1; PD-L1, programmed cell death 1 ligand 1; ROS, reactive oxygen species; TNF, tumor necrosis factor. *Image created with BioRender.com*.

The traditional view of neutrophils as exclusive fast-responders and microbicidal effectors has been challenged by the identification of their capacity to release soluble mediators and NETs as well as cell-derived vesicles and thereby modulate the responsiveness of cells in their vicinity or at distance [149]. The interaction partners of neutrophils differ in TB with infection stage, tissue site and lesion zonation. During early TB, neutrophils interact with lung-residing cells, notably alveolar macrophages, mucosal dendritic cells and pneumocytes, as well as with recruited monocytes and platelets [5,6,115,150]. Once infection is established, they network in addition with lymphocytes (Figure 2). Neutrophils drive granuloma formation by signaling through CXCR3 ligands [151] and thus orchestrate tissue remodeling. Neutrophils’ cross-talks within TB lesions largely depend on the existing microenvironments, with some factors, such as hypoxia, extending their viability and altering their activation mode, e.g., degranulation and inflammatory profiles [125].

### 3.1. Macrophages

Macrophages and neutrophils bidirectionally cross-talk via cytokines and transfer of intracellularly stored molecules, mycobacterial antigens or replication-competent bacteria. *Mtb*-infected macrophages release CXCR2 ligands, primarily CXCL1 and CXCL2, which recruit neutrophils during acute TB in mice [7]. Moreover, *Mtb* replication in mononuclear phagocytes and their necrosis regulate the pulmonary accumulation of neutrophils [152]. Human alveolar macrophages release IL-8 following *Mtb* infection or upon stimulation with LAM, LM, and PIM, indicating a similar role in driving the early accumulation of neutrophils at the infection sites [153]. At late TB stages, neutrophils may govern their lung dynamics via cell-intrinsic mechanisms. For instance, granule-stored S100A8/A9 increases the cell surface abundances of the integrin CD11b and positively regulates neutrophil accumulation [13,154]. On the contrary, miR-223, which is enriched in neutrophils, limits recruitment by the post-transcriptional regulation of CXCL2 [155]. Neutrophil-stored leukotriene B4 or specialized pro-resolving lipid mediators, including lipoxins, resolvins, protectins, and maresins, derived from infected macrophages or neutrophils, may also modulate the tissue dynamics of neutrophils in TB.

Direct interactions between macrophages and neutrophils affect the fate of *Mtb*. Human necrotic *Mtb*-infected neutrophils promote bacillary replication in macrophages [19]. On the contrary, the passive transfer of azurophilic granules or granule-stored NE and MPO [73], as well as apoptotic blebs [74,156], restrict *Mtb* replication in human macrophages. Reductions in *Mtb* growth have also been found in guinea pig alveolar macrophages fed with apoptotic-infected neutrophils and depended on the induction of TNF-α and IL-1 [157,158]. Extracellular vesicles from infected neutrophils trigger autophagy in macrophages [159], and NETs-derived LL-37 in complex with DNA limits *Mtb* replication when taken up by human macrophages [160]. These disparate observations indicate that neutrophils are potential resistance effectors, presumably in early infection, yet they also act as multiplicators of infection, presumably late in TB or in a context-dependent manner. The exchange of particulate material, such as granule content, blebs or vesicle, rather than soluble factors, diminishes the replication of *Mtb* within permissive macrophages.

### 3.2. Dendritic Cells

The cross-talk of neutrophils with dendritic cells (DCs) encompasses direct, contact-dependent events as well as paracrine signaling via cytokines. Neutrophils directly shuttle *M. bovis* BCG [161] or promote the trafficking of *Mtb* to the draining lymph nodes and thus facilitate the priming of naïve T-cells [8,162]. They provide DCs with mycobacterial antigens, facilitate DC chemoattraction by release of CCR7 ligands, possibly CCL19 and CCL21, and prevent *Mtb* inhibition of DC migration to the lymph node [162]. In line with observations in mice, human DCs are activated by the detection of apoptotic *Mtb*-infected neutrophils. This process involves cross-talks via CD11b/CD18 integrins on the neutrophils and DC-SIGN signaling on the DCs [163]. Murine and human neutrophils contribute to the cross-presentation of mycobacterial antigens by DCs [164]. Mechanistically, the cross-presentation involves close contact and a boost in DC activation by neutrophils. In addition, the phagocytosis of apoptotic *Mtb*-infected neutrophils by immature DCs triggers the proliferation of CD8^+^ lymphocytes in a process that requires CD36, but not DC-SIGN [165]. Thus, molecular determinants of DCs differentially regulate their interactions with neutrophils, whereas the chemokines released by neutrophils and their cell fate modulate the antigen presentation capacities and the migratory features of the DCs.

### 3.3. Platelets

Platelets outnumber any leukocyte population in the blood and their roles extend beyond clotting in host defense [166]. Thrombopoiesis occurs in the lung [167], the networking of neutrophils with platelets has been identified in various pulmonary pathologies [166] and neo-vascularization accompanies TB lesions [168]. Accordingly, immune cells could interact with platelets in TB at various stages of the disease. Aggregates of platelets with neutrophils are present in the bronchoalveolar space of *Mtb*-infected mice and have also been detected in the blood of TB patients [150]. Platelets leave neutrophil kinetics, their *Mtb* content, viability and NET formation unchanged. However, platelets limit ROS abundances in neutrophils and thus ultimately contribute to *Mtb* replication in the lung. Whether thrombocytosis and platelet activation, as observed in TB patients [169,170], is controlled by neutrophils remains to be established.

### 3.4. Alveolar Epithelial Cells

Pneumocytes modulate lung cell dynamics via the release of chemokines. *Mtb* moieties stimulate TLR2 on alveolar epithelia for the release of CXCL5 and early neutrophil recruitment [7]. However, TLR2 seem to curtail CXCL5 production upon infection with the W-Beijing strain HN787 [171], which indicates a more complex regulation of chemokine release in pulmonary epithelia. In line with this, the secreted virulence factor ESAT-6 triggers IL-8 production in human bronchial cells and type II pneumocytes and the release of CXCL1 in mice upon lung instillation [172]. Thus, multiple mycobacterial molecular patterns control the production of neutrophil chemoattractants and this redundancy may indicate an essential role of the cross-talk between epithelia and neutrophil for TB outcome. After the generation of adaptive immunity, IL-17A-sensing by non-hematopoietic cells elicits the production of CXCR2 agonists, notably CXCL1 and CXCL5, which further regulate neutrophil recruitment [6]. By producing alveolar lining fluid, type II pneumocytes remodel the *Mtb* cell wall and alter its interactions with neutrophils [122]. Whether neutrophils alter the functions of pulmonary epithelia remains undefined. The transmigration of neutrophils causes a localized depletion of oxygen at mucosal sites [173] and this scenario is likely to happen in TB. Furthermore, neutrophil granular proteins and hydrolases as well as neutrophil-derived ROS and RNS could also impact on the biology of lung-residing cells.

### 3.5. Lymphocytes

Neutrophils accelerate the activation of antigen-specific T-cells in TB, which is largely a consequence of their interaction with DCs [8,162]. During chronic TB, neutrophils rather shut down adaptive immunity through the release of regulatory cytokines or the upregulation of inhibitory receptors. Such regulatory effects may circumvent collateral tissue damage at the expense of antimicrobial immunity. Neutrophils from *Mtb*-infected macaques inhibit antigen-dependent IFN-γ release by T-cell *ex vivo*. However, neutrophil cytokine patterns, e.g., IL-10 positivity, in lesions do not correlate with cytokines immunostained in T-cells, for instance IFN-γ or IL-4 [47]. In *M. bovis* BCG-challenged mice, neutrophils employ IL-10 to shut down IL-17 production by CD4^+^ T-cells [174]. In TB patients, the circulating neutrophils present abundant programmed death ligand 1 (PD-L1) on their cell membrane [175]. The upregulation of PD-L1 and its potential interactions with its cognate receptor PD-1 on T-cells could dampen exuberant inflammation. Indeed, PD-1-mediated inhibition is critical for *Mtb* control in macaques, and PD-1 blockade promotes TB reactivation [176], yet a role for neutrophils as negative regulators awaits validation. Subsets of neutrophils termed granulocytic myeloid-derived suppressor cells inhibit the proliferation of T-cells as well as the release of cytokines in *Mtb*-infected mice [177,178]. Circulating and lung-residing suppressive neutrophils, including LDGs, have also been identified in TB patients [179,180,181].

T-cells modulate through cytokines the *in situ* viability of neutrophils during TB. IFN-γ inhibits the production of IL-17 by CD4^+^ T-cells, thereby limiting neutrophil recruitment. In addition, IFN-γ impairs neutrophil survival in the lung [23]. Moreover, NO, which is induced by IFN-γ, limits neutrophil recruitment by repressing an IL-1- and 12/15-lipoxygenase-dependent chemotactic cascade [20]. However, NK cell-derived IFN-γ drives anti-mycobacterial activities in neutrophils independently of T-cells, suggesting a compensatory function for neutrophils in the absence of IFN-γ [182].

B-cells regulate neutrophilia by modulating the IL-17 response in TB [183]. They also downregulate neutrophil motility with positive effects on abundances of IFN-γ producing T-cells after *M. bovis* BCG vaccination [184]. Neutrophils contain the B-cell-activating factor (BAFF) and a proliferation-inducing ligand (APRIL) and employ both to modulate the immunoglobulin class switching, somatic hypermutation and antibody production in marginal zone B-cells in the spleen [185]. Their precise impact on antibody quality in TB has not been evaluated.

The contribution of other cell types (innate lymphoid cells, MR-1-restricted T-cells) and distinct cell subsets (cDC, pDCs, classical and non-classical monocytes) to TB pathogenesis is currently being established. The timings, molecular determinants and outcomes of dialogues between neutrophils and such “new” interaction partners, along with additional details on their cross-talk with “old” network partners, will enable the assembly of a neutrophil interactome in TB.

## 4. The Neutrophil *In Vivo*: Lessons Learnt from TB Disease Models

Several experimental animal models have been used to pin down the roles of neutrophils in TB. These models, including rodents, non-human primates and cattle complement each other and have been employed to answer distinct scientific questions.

### 4.1. Mice

Mice are frequently used to investigate the functions of immune cells in TB [186]. Inbred mouse lines, transgenic strains and more recently diversity outbred mice [21] as well as CC lines [187] have provided useful insights into the biology of neutrophils upon *Mtb* challenge. Comparing TB-resistant mouse strains such as C57BL/6, A/Sn and Balb/c and TB-susceptible ones, including I/St, 129S2, DBA/2 and C3HeB/FeJ, the connection of neutrophil abundances with disease susceptibility became obvious [22,24,144,188,189]. Using depletion experiments, a detrimental role of neutrophils in progressive TB could be demonstrated. By applying monoclonal antibodies targeting the cell surface epitope Ly6G, a neutrophil marker, the effects of neutrophils in various mouse strains and at distinct stages of TB could be established. Neutrophil depletion alters the overall outcome of TB solely in genetically susceptible mice. This intervention reduces lung pathology and bacillary burden [22,144,188]. The frequencies of IFN-γ-producing cells specific for mycobacteria were inconsistently higher in neutrophil-depleted mice [22,24,188,190]. Contradictory to observations in aerogenic *Mtb* infection, the depletion of neutrophils during the first week after intravenous challenge leads to enhanced *Mtb* replication in infected organs, possibly due to lower NO abundance and the reduced expression of IFN-γ in the tissue [191]. Of note, these effects could be related to the depletion of other myeloid populations, e.g., monocytes and macrophages, in addition to neutrophils because the antibody used for depletion targets also the Ly6C epitope in addition to the Ly6G epitope (clone RB6-8C5). Nevertheless, neutrophil apoptosis during TB contributes to DC activation and the priming of naïve CD4^+^ T-cells [8,148], which are major producers of IFN-γ and subsequently NO, which is a potent anti-mycobacterial molecule. However, despite increasing cell frequencies of antigen-specific CD4^+^ lymphocytes, the effects of neutrophils on *Mtb* load are negligible in low-dose aerosol infection [8]. Thus, the *Mtb* entry sites, e.g., pulmonary versus systemic infection, as well as the dynamics of infection, e.g., dose and organ spread, may be affected differently by neutrophils.

Mice have been instrumental in clarifying the molecular pathways guiding neutrophil recruitment to the lung, e.g., by chemokines and cytokines, and also for deciphering mechanisms controlling the tissue turnover of the neutrophils. Using knock-out strains and cytokine neutralization studies, the roles of CXCR2 and its ligands CXCL1, 2 and 5 [7,171], as well as of the S100A8/9 proteins [13,154], have been established for early and late neutrophil accumulation, respectively. Cell-transfer investigations along with genetic ablation have unveiled that IFN-γ restricts the influx of neutrophils into infected lungs by decreasing IL-17 production and through a direct effect on neutrophil viability [23]. Certain murine models bear neutrophil-intrinsic differences; for instance, cells from I/St mice show a lower expression of the apoptotic receptor CD95, a higher phagocytic capacity, and an increased mobility compared to A/Sn mice [144]. The identification of neutrophil-intrinsic features linked to either resistance or susceptibility to pulmonary TB contributes to a better understanding of disease pathogenesis and the mouse model offers multiple investigational tools in this respect. Discrepancies between murine and human neutrophils, e.g., blood abundances, the expression of arginase 1 [192], defensins [193], need to be carefully considered when extrapolating findings from murine models to human TB.

### 4.2. Guinea Pigs

A unique feature of guinea pigs infected with *Mtb* is the development of lesions that remarkably well resemble human granulomas [194]. In this model, early inflammatory events occurring at day 15 post-challenge [195] encompass an influx of MIL4^+^ cells, notably eosinophils and neutrophils. This inflammatory pattern has also been detected in other tissues, e.g., spleen and lymph nodes [196]. In connection with the elevated neutrophil influx, necrotic lesions are detectable already at day 20 post-challenge [195]. The abundance of neutrophils increases in the lungs as TB progresses and correlates with a more severe pathology [196]. NETs are detected in guinea pig lesions and they have no anti-mycobacterial effect [114]. During chronic TB, the frequencies of CD4^+^ T-cells decrease and B-cells as well as granulocytes populate the lesions, replacing the CD4^+^ lymphocytes [196]. Interestingly, the neutralization of TGF-ß reduces lymphocyte abundance in a guinea pig model of TB pleurisy and triggers a CXCL-8 driven neutrophil influx [197]. The accumulation of neutrophils therefore marks an imbalanced immune response to *Mtb*. High numbers of granulocytes can be found within necrotic lesions [198,199] and, as in humans, neutrophils are localized at the necrotic core and contain S100A9 proteins [200] that likely contribute to tissue damage. Beside the negative effects of neutrophils on granuloma caseation, in vitro studies with guinea pig neutrophils suggest beneficial early events. The phagocytosis of apoptotic *Mtb*-loaded neutrophils by alveolar macrophages leads to the suppression of mycobacterial growth [157]. These observations await *in vivo* validation.

### 4.3. Cattle

Cattle can be naturally infected with *M. bovis* [201], but seem resistant to disease caused by *Mtb* [202]. Infection with *M. bovis* leads to pulmonary pathology resembling TB lesions in humans [202]. Neutrophils populate innate granulomas in animals inoculated intranasally [11] and are detected in low to moderate numbers at day 30 post-challenge in mature granulomas [10]. Within these lesions, the necrotic areas are surrounded by neutrophils [203]. *M. bovis* challenge induces an upregulation of neutrophil-related genes [204] and IFN-inducible genes, e.g., *CXCL10*, *CD274* or *STAT1* [205]. This is similar to the IFN signature detected in neutrophils in human active TB [25]. In contrast to other species, the number of lymphocytes in blood increases with TB progression, whereas the number of neutrophils decreases. One possible explanation is the downregulation of *IL8*-related genes and the subsequently lower IL-8 release, a key mediator for neutrophil recruitment [206]. The ex vivo exposure of bovine neutrophils to *M. bovis* induces necrosis and cells fail to eliminate bacilli, unless starved and activated by TLR-ligands [207]. These features resemble the responses of human neutrophils to *Mtb* infection. The responses of cattle neutrophils to *Mtb* and *Mb* have not yet been investigated comparatively to establish the potential roles of these phagocytes in the resistance of cattle to *Mtb*.

### 4.4. Non-Human Primates

Cynomolgus and rhesus macaques are widely used to study immunity to infection, as both species reflect the immunoreactivity of humans. The exposure of cynomolgus macaques to low doses of *Mtb* results in outcomes similar to those observed in humans: clearance of infection, fast-progressing disease, or latent TB and disease reactivation [9,208]. The neutrophil influx into the lung is accelerated in LTBI macaques compared to LTBI humans [209]. Animals developing active TB show a broad spectrum of different granuloma types: caseous, solid and neutrophilic granulomas with remarkable neutrophil accumulations [210]. Neutrophils purified from *Mtb*-challenged cynomolgus macaques secrete TNF-α, IL-4, or IL-10 upon stimulation with mycobacteria and restrict antigen-specific production of IFN-γ by T-cells. Although IFN-γ has been scantly detected in peripheral neutrophils, cells within granulomas exhibit a strong immunostaining for this cytokine as well as for IL-10, unlike their human counterparts [47]. Neutrophils of cynomolgus macaques express granzyme B, a pro-apoptotic enzyme that is associated with cytotoxicity, in granulomas. *In vitro*, granzyme B lacks bactericidal activity. However, granuloma containing a higher mycobacterial burden show more granzyme B-positive neutrophils, whereas reduced granzyme B-positive neutrophils were associated with lower bacterial burden [211]. These findings in non-human primates (NHP) indicate that neutrophils are endowed with immunoregulatory features and that they modulate the microenvironment of TB lesions.

Each animal model has unique advantages for the study of TB pathogenesis. The mouse model remains the most widely used one for investigations focusing on immunity [186]. The availability of inbred and transgene mice, as well as of multiple immunological tools such as depleting antibodies, and reagents for immunophenotyping, cell tracking and fate mapping, allow for mechanistic studies. Natural hosts, e.g., cattle or NHP, provide insights into TB progression, lesion composition and natural resistance. However, for certain models, including guinea pigs and cattle, the immunological toolbox is rather basic and breeding and housing is expensive. Moreover, certain disease mechanisms and TB progression may differ from the processes and events in humans with active TB [194]. Hence, although each model offers valuable insights into TB pathophysiology, caution should be taken regarding the generalization of findings.

## 5. Perspectives: The Neutrophil in TB Today and Tomorrow

The neutrophil research is currently enjoying a revival. Single cell analyses, cell fate mapping, advanced *in vivo* imaging and transgene technologies advance current knowledge about neutrophil biology. Neutrophils are phenotypically diverse, regulate organ homeostasis and have functions extending beyond microbial killing [212]. Today’s neutrophil facts and their emerging functions spark new ideas in TB research. The heterogeneity of neutrophils has been documented at steady state as well as during acute and chronic inflammation [213]. The impact of the heterogeneity of neutrophils on TB progression, bacterial dissemination and lesion resolution has been scantly addressed so far. Are all neutrophils equal in supporting permissiveness to bacteria, or triggering tissue damage? Novel data indicate that long-lived Ly-6G^dim^ neutrophils are more permissive for *Mtb* replication [190] and suppressive neutrophils, including low-density neutrophils and granulocytic myeloid-derived suppressor cells, are associated with exuberant inflammation [177,178,181]. Neutrophils imprint on tissue homeostasis [214]. Does neutrophil tissue imprinting affect TB outcome in various organs? Neutrophils undergo innate training and epigenetic rewiring of granulopoiesis as well as neutrophil reprogramming could benefit cancer therapy [215]. Do mycobacteria directly or indirectly interfere with the trained immunity of neutrophils? Interference with trained immunity through IFN-I signaling coupled to iron metabolism and the specific loss of myeloid progenitors has been described for *Mtb* [216]. *M. bovis* BCG induces long-lasting functional changes in neutrophils of human vaccinees by causing chromatin remodeling [217] and a bias towards granulopoiesis in the hematopoietic stem and progenitor cells has been observed upon *M. bovis* BCG vaccination in mice and humans [218,219]. Whether exposure to *Mtb* or recovery from TB causes reprogramming in neutrophils remains to be evaluated. The transcriptional responses of neutrophils are sex-biased, with signatures of hyperresponsiveness to IFN-I in females and increased mitochondrial metabolism as well as immaturity features in males [220]. Do these sex differences contribute to the male bias observed in TB susceptibility? The neutrophil-driven IFN-I signature characterizes TB [25], yet whether sex preconditioning alters this critical pathogenic trait of TB is not known. Finally, in spite of technical advances facilitating population screens in experimental models, a precise definition of subcellular events within neutrophils remains elusive. Finding answers to these questions will offer refreshed views on TB pathophysiology and will improve disease control measures. Certainly, neutrophils will keep on teaching us about TB in the future.
